# Traveling waves along the cortical depth reflect structured synaptic inputs

**DOI:** 10.21203/rs.3.rs-10385703/v1

**Published:** 2026-08-26

**Authors:** Lihao Yan, Mitchell Morton, Anirvan S. Nandy, Monika P. Jadi

**Affiliations:** 1 Department of Physics, Yale University, New Haven, CT 06520; 2 Department of Psychiatry, Yale University, New Haven, CT 06510; 3 Interdepartmental Neuroscience Program, Yale University, New Haven, CT 06510; 4 Department of Neuroscience, Yale University, New Haven, CT 06510; 5 Department of Psychology, Yale University, New Haven, CT 06511; 6 Kavli Institute for Neuroscience, Yale University, New Haven, CT 06511; 7 Wu Tsai Institute, Yale University, New Haven, CT 06511

## Abstract

Traveling waves (TWs) are widely observed in the brain and are thought to play multiple functional roles, yet what LFP traveling waves represent remains unclear. Using translaminar recordings in macaque V1 and V4, we observed local field potential (LFP) TWs that were modulated by sensory experience, but no corresponding TWs in the multi-unit activity envelope (MUAe). Nevertheless, LFP TW periods were associated with increased depth synchrony of MUAe. Crucially, TW speeds predicted population spiking dynamics: faster waves were associated with larger MUAe amplitude, whereas slower waves are associated with smaller MUAe amplitude. We propose that LFP TWs reflect spatiotemporally structured excitatory inputs, while MUAe reflects their leaky integration. This framework provides a parsimonious explanation for both the observed traveling waves and their relationship to population spiking, offering a physiological interpretation of laminar LFP traveling waves, in which TW speed reflects the temporal organization of structured synaptic inputs.

## INTRODUCTION

Neural oscillations, or rhythmic fluctuations of neural activity, have been a key focus in neuroscience since their discovery^1^. Neural oscillations have been implicated in a range of important cognitive functions, including sleep^2^, perception^3^, spatial navigation^4^, attention^5^ and memory^6^. For example, 4–8 Hz (“theta band”) oscillations have been shown to play a functional role in learning^7^, while 8–13 Hz (“alpha band”) oscillations predict the temporal resolution of visual perception^8^.

Neural oscillations have traditionally been studied by averaging across trials, which can obscure meaningful patterns in the signal^9,10^. Recent advances in dense multi-electrode arrays have enabled recordings with high spatiotemporal resolution and signal-to-noise ratio^11^, making single-trial analysis more reliable. Such analyses show that, for example, previously observed 2–15 Hz oscillations in humans are traveling waves (TWs)^12,13^, which are patterns of neural activity characterized by a systematic phase-shift in cortical space. TWs have been linked to sensory^14–22^, motor^23,24^, and memory^25–27^ processes, where they have been implicated in perceptual gating^14^, movement initiation^23^, stimulus encoding^20^, and reward history^25^. In addition, they have been observed in developmental processes^28–30^ and pathological conditions^31–33^. More broadly, they have been widely observed in multiple brain regions ^10,14,17,19,24,34–38^ and species^10,12–15,17,19,23–25,30,37–42^.

Despite growing evidence for cortical TWs across species, brain regions, and recording modalities, a fundamental mechanistic question remains unresolved: what do traveling waves observed in extracellular field potentials represent? While TWs have been observed in multiple modalities, including functional magnetic resonance imaging^39,43^, local field potentials (LFP) ^13,14,17,19,21,24–26,37,44^, magnetoencephalography^45,46^, voltage-sensitive dyes^10,16,41,42^ and calcium indicators^47–49^, these signals do not directly reflect action potentials, and it therefore remains unclear whether the observed TWs reflect the spatiotemporal dynamics of synaptic *inputs* or spiking *outputs* of the cortical networks^11,50,51^. Resolving this distinction is essential because it fundamentally changes the interpretation of TW dynamics. For example, the physiological interpretation of TW speed depends fundamentally on the underlying mechanism: it could either reflect the temporal organization of synaptic inputs or the propagation speed of neural outputs.

The columnar population of the neocortex provides the minimal setup for studying TWs, with its spatial bounds, shared functional tuning, stereotypical connectivity^52–54^ and segregated input targets along the depth^55^. Consequently, it offers a unique axis along which synaptic inputs (reflected in local field potentials) and population outputs (reflected in neural spiking activity) are separable yet coupled, enabling direct mechanistic investigation of the nature of TWs. Despite these structural advantages, it remains unknown whether and how single-trial TWs propagate along the cortical depth.

In this study, we investigated TWs along the cortical depth using laminar recordings from macaque visual cortical areas V1 and V4. We found that TWs are organized robustly in LFP but absent from the multi-unit activity envelope (MUAe). Instead, LFP TW speeds predict the magnitude and synchrony of population spiking. We propose a mechanistic framework in which LFP TWs reflect the spatiotemporal organization of synaptic inputs, while MUAe reflect their leaky integration within cortical columns. This framework provides a physiological interpretation of LFP traveling waves, links extracellular field dynamics to neuronal output, and generates experimentally testable predictions.

## RESULTS

### LFP TWs occur robustly across sensory experiences

**Figure 1.**
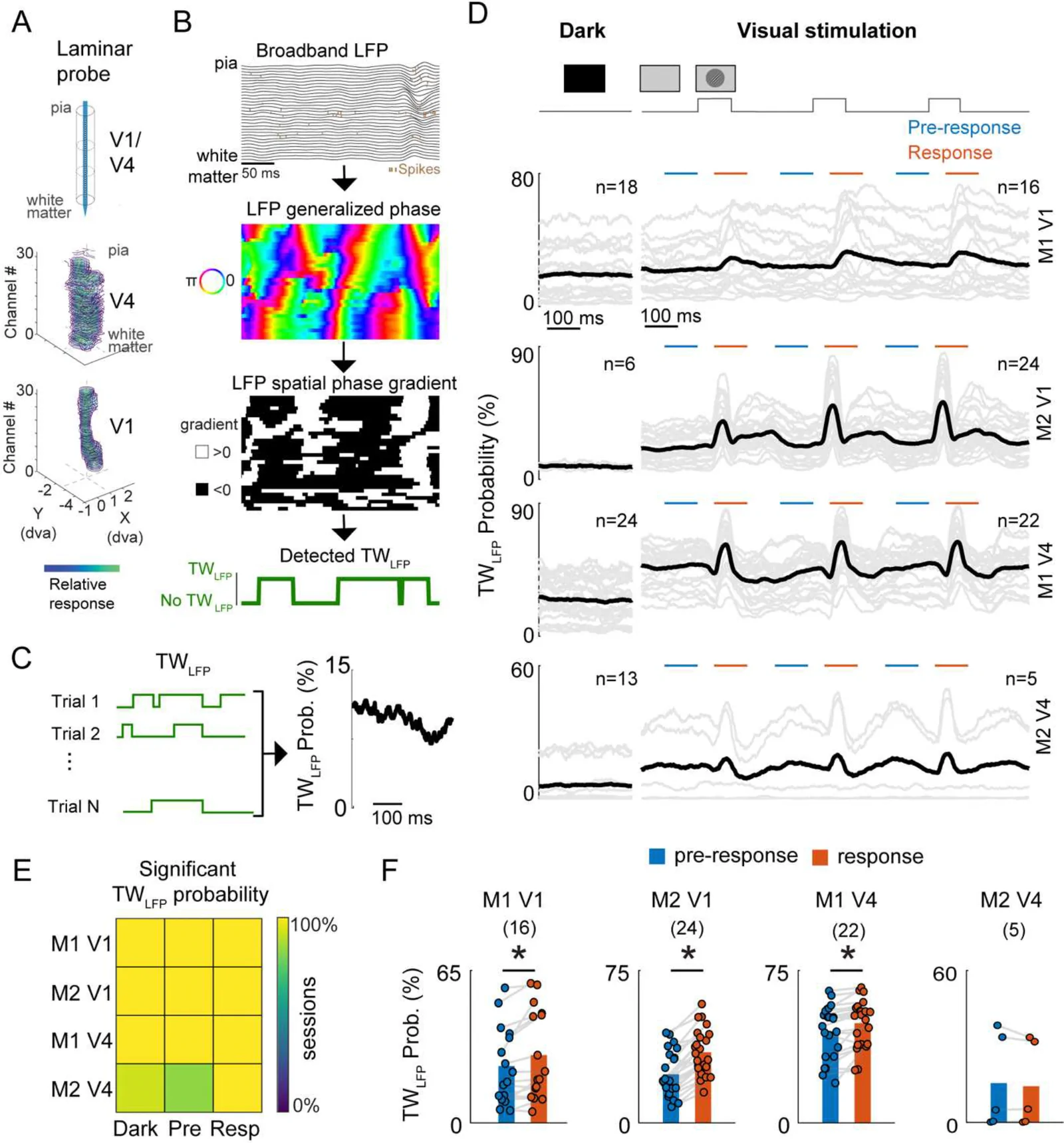
Modulation of LFP traveling wave probability by sensory experience. (**A**) LFP-based spatial receptive fields from example translaminar recording sessions from areas V1 and V4, showing columnar sampling of activity. dva: degrees of visual angle (**B**) Analysis steps for detecting TWs in broadband local field potential (LFP) recorded along the cortical depth using linear array probes. (**C**) Estimation of the temporal evolution of LFP TW probability using time-locked “trials”. Trials were of fixed period, time-locked either to voluntary saccade onset during spontaneous viewing in darkness or to stimulus onset during periodic presentation of oriented Gabor patches during fixation (see Methods). (**D**) Temporal evolution of LFP TW probability during dark periods and visual stimulation. n indicates the number of sessions. Black line represents session-averaged probability, and gray line represents single session traces. Blue and red bars mark the pre-response and response periods. TW probability evolution with multiple stimuli is shown for illustration only. The remainder of the analysis for visual stimulation period is presented with trials defined around single stimulus presentation. (**E**) Percentage of sessions with significant LFP TW probability. Significance was established using one-tailed t-test, p<0.05. Session counts are provided in Table 1. (**F**) Comparison of average LFP TW probability between pre-response and response periods. Numbers in parentheses indicate session counts. Bars represent the average LFP TW probability across sessions; each dot represents the pre-response or the response period in a session, with lines connecting those from the same session. p<0.05, one-tailed pairwise Wilcoxon signed rank test.

To investigate the prevalence of traveling waves (TWs) along the cortical depth, we collected and analyzed LFP data from laminar multi-electrode arrays in macaque visual cortical areas V1 and V4 (Fig. 1A, previously unpublished, except partial V1 data^56^. See Methods). We examined data from two categories of sensory experiences: dark periods with no visual stimuli, and visual stimulation periods with periodic presentations of Gabor patterns inside or near the receptive field of the recording site during fixation.

To robustly detect putative TWs, we used a single-trial analysis pipeline that estimated periods of significant spatial and temporal coherence of the generalized phase (GP) gradient of LFPs along the depth (Fig. 1B and Methods). TW probability was estimated as the fraction of recording duration in a session that exhibited putative TWs (Fig. 1C). When we sampled time-locked, fixed-duration blocks during both sensory experiences (Methods), the temporal evolution of LFP TW probability indicated robust occurrence under both conditions (Fig. 1D).

To quantitatively assess the existence of LFP TWs in both experiences, we identified three periods of interest—dark, pre- visual response, and visual response—and compared the empirical TW probability with null distributions obtained from both the temporally shuffled data and the spatially shuffled data during these periods (Fig. 1E). Compared to the shuffled data, LFP TW probability was significant for nearly all the periods in all sessions across all four datasets (Fig. 1E; 196/198 session-periods). While LFP TW probability remained relatively stable during the dark period, it was modulated during Gabor stimulation (Fig. 1D).

To quantitatively analyze the modulation during visual stimulation, we compared the average LFP TW probability of each session during the pre-response and response periods (Fig. 1F), and found that it increased during the response period for three datasets (M1’s V1, V4, and M2’s V1), except for one dataset (M2 V4) where insufficient data were available (p-values: M1 V1: 0.007368; M2 V1: 0.000013; M1 V4: 0.000654; M2 V4: 0.906250, one-tailed pairwise Wilcoxon signed rank test).

### LFP TW speed is modulated by sensory experience

**Figure 2.**
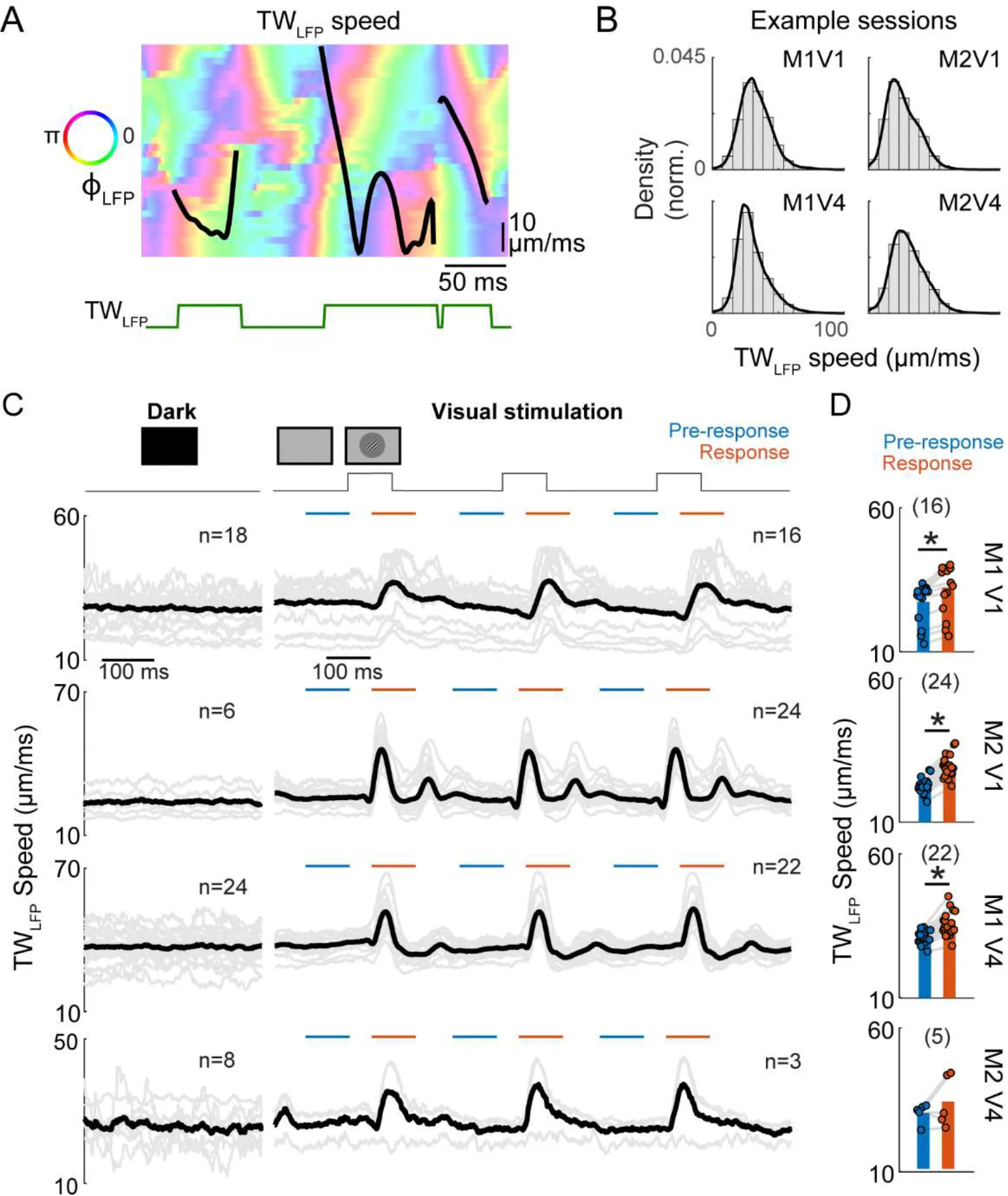
Modulation of LFP traveling wave speed by sensory experience. (**A**) Estimated LFP TW speeds for an example trial, with LFP generalized phase and TW detection periods for reference. (**B**) Histogram of estimated LFP TW speeds for example sessions. (**C**) Temporal evolution of LFP TW speed. n indicates the number of sessions. Black line represents session-averaged speed and gray line represents single session. Blue and red bars mark the pre-response and response periods. (**D**) Comparison of average LFP TW speed between pre-response and response periods. Bars represent the average LFP TW speed across sessions; numbers in parentheses indicate the number of sessions. Each dot represents the pre-response or the response period in a session, with lines connecting those from the same session. p<0.05, one-tailed pairwise Wilcoxon signed rank test.

Prior studies have shown that traveling wave speeds along the cortical surface fell within the physiological range of unmyelinated axonal conduction delays^11,17^. We next analyzed the LFPs to test if the waves detected along the cortical depth followed this pattern, and if their speed was modulated by sensory experience. For each trial, we estimated the speed of LFP TWs by taking the ratio between the average spatial and temporal phase gradients across channels (Fig. 2A and Methods). The estimated speeds were on the order of 10–50 *μm/ms* across all sessions and datasets, in line with surface TW speed estimates^11^ (Fig. 2B).

When we estimated the LFP TW speeds in time-locked, fixed-duration blocks during both types of sensory experiences, we found that the speed was relatively constant during the dark period and the pre-stimulation period of the fixation task (Fig. 2C). However, the wave speed increased during responses to visual stimulation (Fig. 2C). Quantitatively comparing the LFP TW speeds during the pre-response and response periods (Fig. 2D), we observed a significant increase during the response periods for all but one dataset where the least amount of data was available (p-values: M1 V1: 0.000107; M2 V1: 0.000010; M1 V4: 0.000022; M2 V4: 0.218750, one-tailed pairwise Wilcoxon signed rank test).

### LFP TW speed predicts cortical excitability

**Figure 3.**
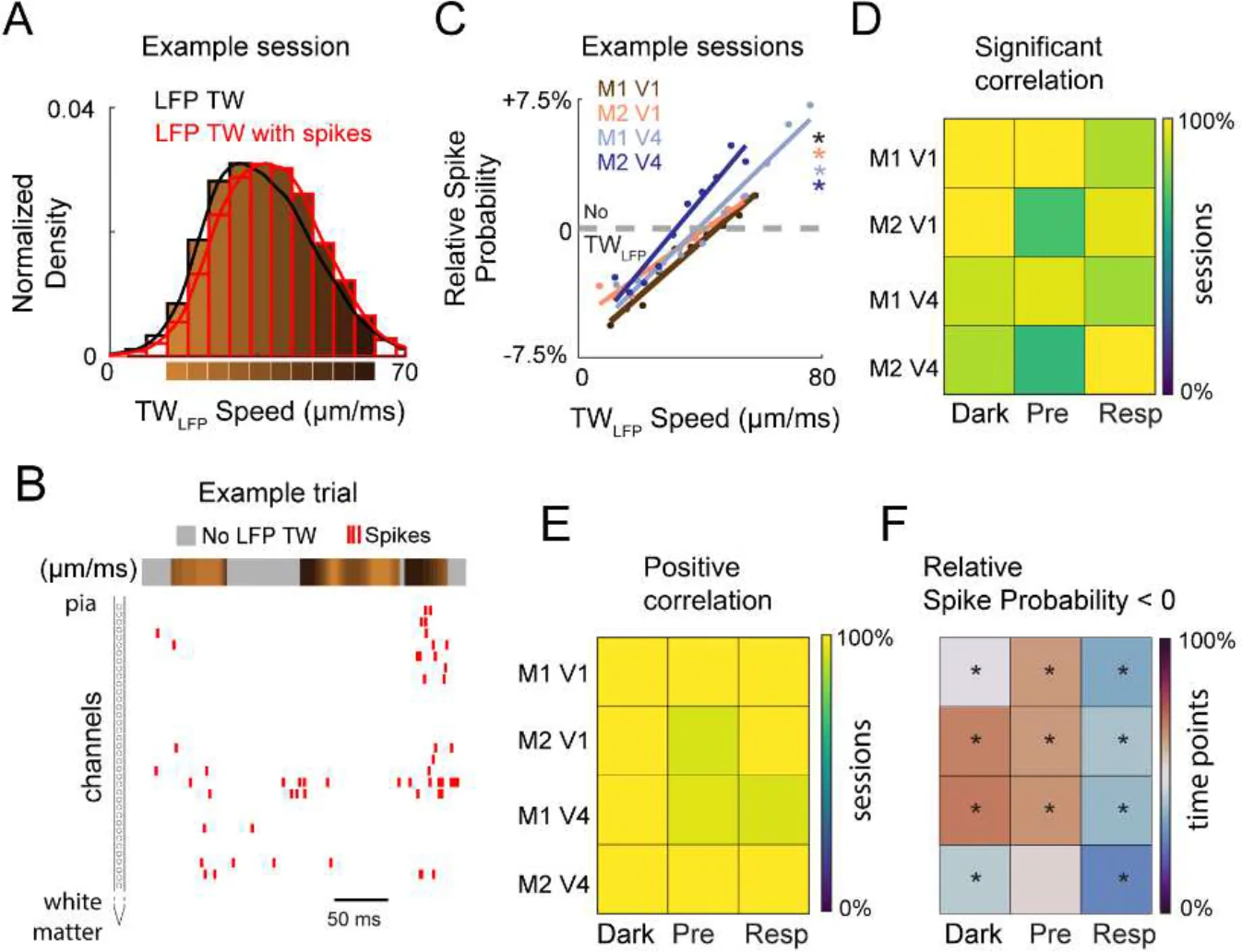
LFP TW speed and excitability. (**A**) Histogram of LFP TW speed with ten speed bins marked in an example session. Black: LFP TW speeds of all time points; Red: LFP TW speeds of time points when spikes were also detected. (**B**) TW speed (top) and spiking activity along the cortical depth for the same trial as Fig. 1A. (**C**) Relative spike probability during periods of LFP TWs compared to no LFP TW periods as a function of TW speed, for example sessions from all four datasets. Each dot represents a speed bin as defined in **A**. The dashed line at zero indicates the average spike probability from periods when no LFP TW was detected; p<0.05, two-tailed t-test. (**D**) Percentage of sessions with significant correlation between LFP TW speed and spike probability. “Dark” denotes the dark period, “pre” the pre-response period, and “resp” the response period. (**E**) Percentage of significantly correlated sessions with positive correlation. (**F**) Percentage of time points with spike probability below the baseline where no LFP TW is detected; p<0.05, two-tailed t-test. Asterisk indicates significance. For (**D**–**F**), session counts are provided in Table 1.

Given that cortical excitability is strongly modulated by sensory experience, we hypothesized that LFP TW speed might correlate with the underlying spiking activity. To test this hypothesis and to characterize the relationship between LFP traveling wave speed and spike probability, we performed a binned analysis of LFP TW speeds (Fig. 3A and Methods). At a single trial level, we observed more spikes at higher speeds than at lower speeds (Fig. 3B), suggesting that spike probability was higher for faster LFP TWs. By estimating spike probability for each speed bin as the fraction of time points in the bin with detected spikes (see Methods), we found that spike probability in example sessions from all four datasets correlated positively with LFP TW speed (Fig. 3C). Notably, spike probability for a substantial portion of LFP TW time points was below that estimated for periods without LFP TWs.

At the single session level across all three sensory experience periods from all four datasets, the correlation between LFP TW speed and spike probability was almost always significant (Fig. 3D, 164/182 session-periods) and positive (Fig. 3E, 161/164 session-periods), and a significant proportion of data was associated with periods of lowered spike probability (Fig. 3F). We observed similar findings when restricting our analysis to either narrow (putative inhibitory) or broad (putative excitatory) single units (Fig. S3A) and to spiking activity in different cortical layers (Fig. S3B).

The lowered excitability was unexpected, as LFP TWs are thought to correlate with the propagation of spiking activity^11,51^. To further understand the relationship between LFP TWs and spiking activity, we next analyzed the multi-unit activity envelope along the cortical depth.

### MUAe lacks TWs but varies with LFP TW speed

**Figure 4.**
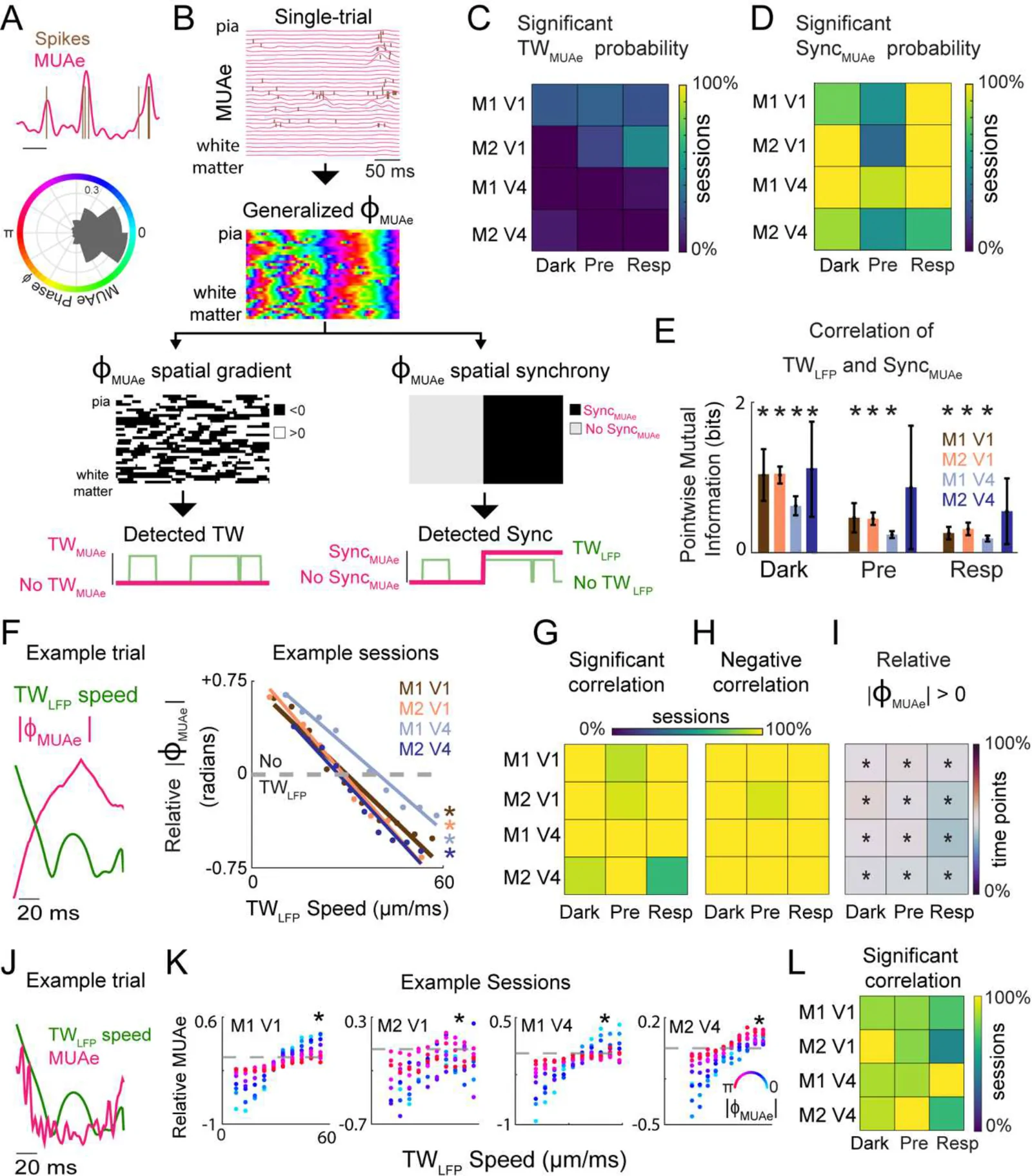
Multi-unit activity envelope (MUAe)-based characterization of spatiotemporal organization of spiking activity in the cortical column. (**A**) *Top*: Example trial showing MUAe fluctuations co-occurring with spike times at a recording channel (see Methods for details). *Bottom*: Histogram of spike frequency as a function of MUAe phase for an example session. (**B**) Analysis steps for detecting TW (left branch) and synchrony (right branch). Example laminar MUAe shown during the same trial as in Fig. 1A and Fig. 3B. Periods of detected LFP TW for the trial (green) are included for reference. (**C**) Percentage of sessions with significant MUAe TW probability. Significance was established using one-tailed t-test, p<0.05. “Dark” denotes the dark period, “Pre” the pre-response period, and “Resp” the stimulus response period. (**D**) Percentage of sessions with significant MUAe synchrony probability. Significance was established using one-tailed t-test, p<0.05. (**E**) Correlation between MUAe synchrony and LFP TW measured by pointwise mutual information (see Methods). Error bars represent the 95% confidence interval; p<0.05, two-tailed t-test. (**F**) Left: Fluctuations in TW speed and channel-averaged MUAe phase during a period of coincident LFP TW and MUAe depth synchrony. Right: Session-wide data showing correlation between LFP TW speed and channel-averaged MUAe phase during depth synchrony compared to periods without LFP TW. The dashed line indicates the baseline phase where no LFP TW was detected; p<0.05, two-tailed t-test. Each dot represents a speed bin, which was used for visualization only. Significance was calculated on unbinned data. (**G**) Percentage of sessions with significant LFP TW speed and channel-averaged MUAe phase correlation. (**H**) Percentage of significantly correlated sessions with negative correlation. (**I**) Percentage of time points with channel-averaged MUAe phase greater than that of periods without LFP TW; p<0.05, two-tailed t-test. Asterisk indicates significance. (**J**) Fluctuations in TW speed and channel-averaged MUAe during a period of coincident LFP TW and MUAe depth synchrony. (**K**) Example sessions showing correlations between LFP TW speed and channel-averaged MUAe for various phase values during depth synchrony; p<0.05, one-tailed Seasonal Kendall test. The dashed line at 0 indicates the baseline MUAe where no LFP TW was detected. Each dot represents a speed bin for visualization only; significance was calculated using unbinned data. (**L**) Percentage of sessions with significant positive correlation between LFP TW speed and MUAe. For panels C–E, G-I, and L session counts are provided in Table 1.

To investigate whether LFP traveling waves correlated with propagation of spiking activity, we applied our TW analysis pipeline to the broadband multi-unit activity envelope (MUAe), a high-frequency component (> 500Hz) of extracellular recordings (Fig. 4A, see Methods). MUAe is a continuous signal that represents aggregated local spiking activity^57,58^, and whose generalized phase is inversely related to spike probability (Fig. 4A). MUAe provided better spatial resolution for our analysis compared to single-unit spike activity, as spikes were not always isolated on every recording channel. MUAe data for each channel were derived from the same laminar multi-electrode array recordings as the LFP signal (Methods).

To enable direct comparison between LFP TW and MUAe TW, we applied the analysis pipeline with the same parameters as for LFP TW analysis. For the example trials, LFP TW detection did not guarantee a corresponding TW pattern in the MUAe signal (Fig. 4B). Furthermore, MUAe TW probability was insignificant at the single session level in almost all three sensory experience periods across all four datasets, compared to both spatially and temporally shuffled data (Fig. 4C, 166/198 session-periods). These results suggest that LFP TWs do not reflect the propagation of spiking activity along the cortical depth.

Although we did not observe TW patterns in MUAe, we observed periods of MUAe phase alignment across cortical depth (Fig. 4B), suggesting synchronous spiking activity. We quantified the synchrony by identifying epochs of significant generalized phase coherence in MUAe across channels^59^, and tested whether it was significant for each session compared to temporally shuffled data (see Methods). Across all three periods—dark, pre-response, and response—and across animals and areas, it was significant for most of the sessions from all four datasets (Fig. 4D; 161/198 session-periods).

Additionally, we observed overlaps between periods of LFP TWs and MUAe depth synchrony within individual trials (Fig. 4B, Fig. S4A). To study if they were systematically correlated, we estimated the pointwise mutual information between LFP TW and MUAe synchrony. Across sessions and sensory periods, LFP TW significantly correlated with MUAe synchrony in all three periods—dark, pre-response, and response—and across all four datasets, except for the pre-response and response periods of the V4 dataset from M2 (Fig. 4E).

We interpreted these periods of synchrony as regimes of column-wide coherent computations. Within this regime, we next investigated how the properties of LFP TWs correlated with the properties of columnar MUAe. To characterize the relationship between LFP TW speed and excitability, we estimated the channel-averaged MUAe for periods where both LFP TW and MUAe synchrony were detected (Fig. 4B and Methods). Since the generalized phase of MUAe is inversely related to spike probability (Fig. 4A), we hypothesized that either/both of average MUAe phase and amplitude would be correlated with LFP TW speed, as in the case of spike probability (Fig. 3C–E).

We indeed observed a correlation between LFP TW speed and average MUAe phase: faster LFP TWs were associated with lower MUAe phase, and slower LFP TWs with higher average MUAe phase (Fig. 4F). At the single-session level, the correlation was almost always significant (Fig. 4G, 167/171 session-periods) and almost always negative (Fig. 4H, 166/167 session-periods) across all three sensory experiences and all four datasets. Given that we observed a reduced spike probability in a significant portion of LFP TW time points (Fig. 3F), we investigated whether a similar pattern existed in MUAe activity. We found that the average MUAe phase exceeded a baseline – defined as the channel-averaged MUAe phase during periods of MUAe depth synchrony in the absence of LFP TW – for a substantial proportion of MUAe synchrony epochs. This effect was significant across all three sensory experiences and datasets (Fig. 4I).

MUAe amplitude also showed a significant positive correlation with LFP TW speed in individual trials (Fig. 4J). It was higher for faster LFP TW speeds than for slower speeds at each phase (Fig. 4K). Most sessions across all three sensory experiences and all four datasets showed a significant correlation across phases (Fig. 4L, 141/174 session-periods, p<0.05, Seasonal Kendall test).

### A physiological framework linking LFP TWs to neuronal output

**Figure 5.**
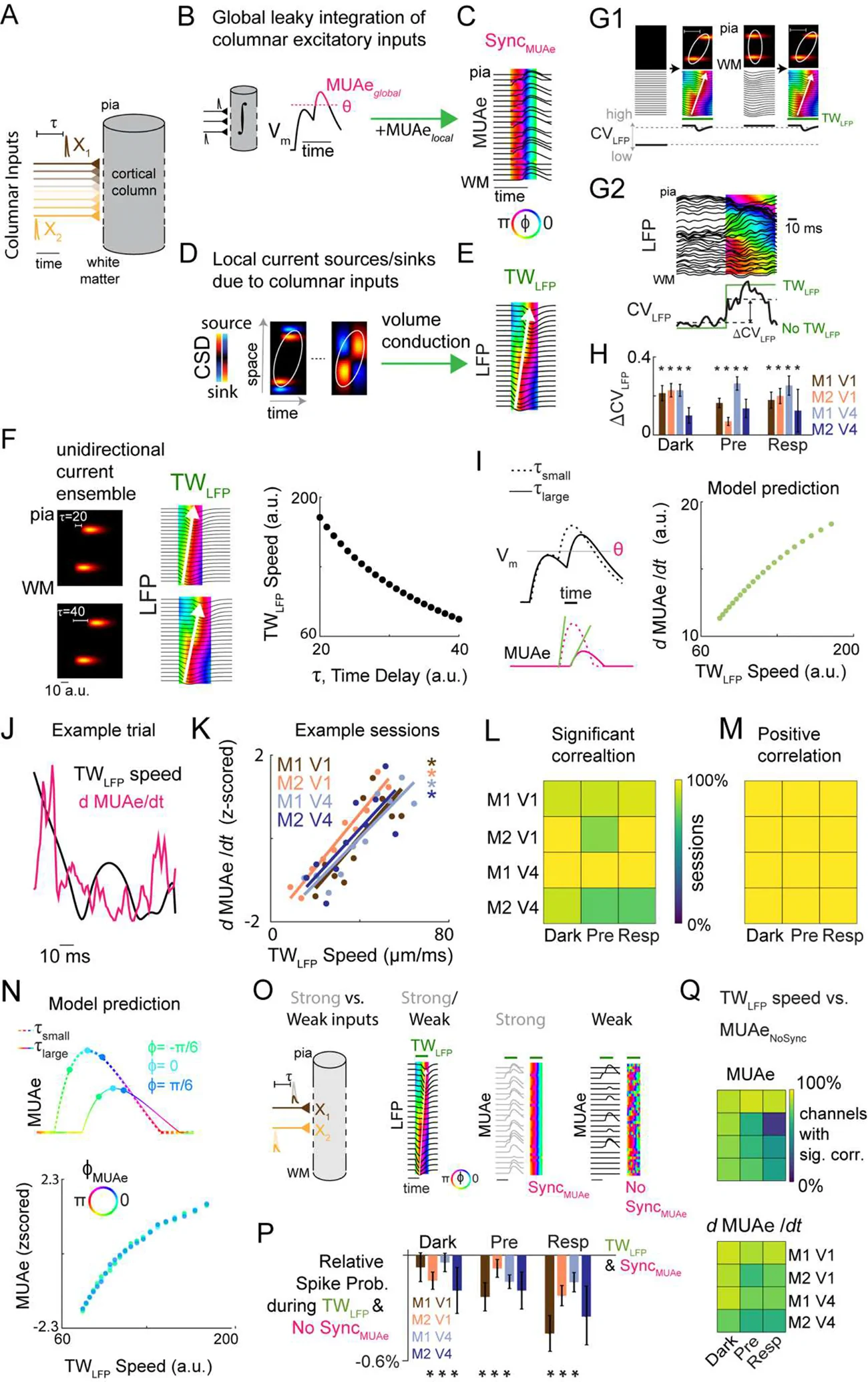
A framework for spatiotemporal organization of local field potential and spiking activity. (**A**-**E**) LFP traveling waves (TW) along cortical depth reflect the spatiotemporal sequence of inputs across layers (A), observed through volume conduction (E) of unidirectional current ensemble patterns (D, marked by white ovals) as a part of evolving dipoles. Leaky integration of these inputs throughout depth is reflected in the local MUAe (B), and hence often detected as synchrony (C). LFP TW, thus, does not reflect propagation of spikes, but the temporal structure of inputs. The spread of input pathways in panel (A) indicates that their physical separation is not a necessary condition for the framework. It is the spatiotemporal organization of the current ensembles or dipoles that determines detection of LFP TWs in this framework. CSD: Current Source Density. (**F**) LFP TW speed as a function of time delays between inputs along cortical depth, as predicted by volume conduction (see Methods). We only consider the simplest unidirectional current ensemble for the modeling results. All results hold when using more complex dipole configurations (for example, Fig S5A). (**G1**) Alternative scenarios for the organization of current ensemble during TW period. Each scenario predicts distinct modulation patterns in LFP variability, measured as coefficient of variation (CV). (**G2**) Single-trial example of modulation of CV during TW. (**H**) Difference in CV between LFP TW and non-TW periods across all sessions in all datasets; p < 0.05, one-tailed paired Wilcoxon signed-rank test. Error bars indicate 95% confidence intervals. (**I**) LFP TW speed (as estimated from volume conduction model) as a function of dMUAe/dt (estimated from leaky integration model). (**J**) Single-trial example of estimated LFP TW speed and estimated dMUAe/dt. Gaussian smoothing was applied for visualization purposes only. (**K**) Example sessions showing correlations between LFP TW speed and dMUAe/dt; p<0.05, two-tailed t-test. Each dot represents a speed bin for visualization only; significance was calculated using unbinned data. Analysis was done during periods of MUAe depth synchrony, and MUAe was averaged across channels before taking the derivative. (**L**) Percentage of sessions with significant correlation between LFP TW speed and dMUAe/dt. (**M**) Percentage of sessions with positive correlation between LFP TW speed and dMUAe/dt. (**N**) LFP TW speed (estimated from volume conduction model) as a function of MUAe magnitude (estimated from leaky integration model). Corresponding phases are color coded. MUAe is z-scored within each phase for illustration only. Inset on the top illustrates MUAe corresponding to fast (dashed) and slow (solid) TWs. (**O**) Prediction for the effect of columnar input strength on MUAe (and its depth synchrony) in the MUAe computational model. TW is detected for both strong and weak exemplar inputs in the LFP computational model. (**P**) Relative spike probability in the translaminar data during periods of LFP TW without MUAe synchrony; p < 0.05, one-tailed paired Wilcoxon signed-rank test. Error bars indicate 95% confidence intervals. (**Q**) Percentage of channels across all sessions with significant correlations between TW speed and MUAe amplitude and slope, during periods of LFP TW without MUAe synchrony. Significance was established using one-tailed Seasonal Kendall test and two-tailed t-test, respectively; p<0.05. For panels L,M,P, and Q session/ channel counts are provided in Table 1.

Our analyses indicate that LFP TWs along the cortical depth do not arise from propagating spiking activity, yet their speed is strongly correlated with the dynamics of population excitability. These empirical dynamics lie beyond the explanatory scope of existing models: some models do not incorporate population spiking^40,50^, and those with neuronal spiking do not incorporate mechanisms for the fast dynamics of TW speed as seen in our data^60,61^. Building on previous work showing that spatiotemporally structured neural activity can generate LFP traveling waves through volume conduction^40,50^, we propose a framework in which synaptic inputs across the cortical depth give rise to LFP TWs through volume conduction, while the same inputs generate MUAe fluctuations through their leaky integration (see Methods, Fig. 5A–E).

Specifically, we propose that inputs targeting cortical depth transiently result in spatio-temporally structured unidirectional (all sources or all sinks) current ensembles (Fig. 5D). Through volume conduction, these ensembles, which can arise from various arrangements of dipoles^50^ (Fig. S5A), are detected as LFP TWs (Fig. 5E). Cortical columns are thought to act as functional units that integrate inputs across the cortical depth through recurrent connections^52–54^. Building on this, we propose that the same synapses inputs that trigger spatio-temporally structured current ensembles, are rapidly shared across the column, likely mediated by strong interlaminar connectivity^52–54^ (Fig. 5B). These shared inputs are integrated throughout the column through a “leaky integration” mechanism (see Methods), in which the output dynamics is sensitive to the temporal alignment of inputs^62^ (Fig. 5B–C). An important implication of this mechanism is that TW speed reflects the temporal organization of the input ensemble. Faster TWs correspond to more temporally aligned inputs, whereas slower TWs reflect greater temporal dispersion (Fig. 5F).

The framework predicts two possible manifestations of structured current ensembles: their emergence, leading to increased spatial variability of LFP signals across depth, or reorganization (Fig. 5G1 Right) with little change in spatial variability of LFP signals. We found that the spatial coefficient of variation (CV) of LFP was significantly higher during TW periods compared to non-TW periods across datasets and conditions (Fig. 5G2–H), suggesting the former scenario.

Within this framework, predominantly excitatory structured inputs provide the most parsimonious explanation for the relationship between input alignment and the MUAe dynamics. This leads to two key predictions. First, we predict that faster TWs should produce steeper MUAe slopes, as temporally aligned excitatory inputs integrate rapidly (Fig. 5I). We found evidence for this prediction across all three sensory experiences and all four datasets: MUAe slope showed a significant positive correlation with LFP TW speed in individual trials (Fig. 5J) and across sessions (Fig. 5K). The correlation was almost always significant at a single-session level (Fig. 5L, 170/179 session-periods) and always positive (Fig. 5M, 170/170 session-periods). Second, we predict that faster TWs should yield larger MUAe amplitudes, as reduced temporal dispersion minimizes leak and increases effective summation of excitatory inputs (Fig. 5N). This prediction was also validated by our empirical observations (Fig. 4J–L).

Finally, this framework also provides a parsimonious explanation for periods in which LFP TW occurs without MUAe depth synchrony (e.g. Fig. 4B). When the TW-generating excitatory inputs are relatively weak, our model predicts a breakdown of synchrony due to dominance of independent local spiking activity along the column (Fig. 5O). Consistent with this, we found that, across most conditions and datasets, spike probability was significantly lower during LFP TW periods without MUAe synchrony compared to those with synchrony (Fig. 5P). Even in the absence of depth synchrony, our framework predicts that LFP TW speed should relate to channel MUAe dynamics, which we observed robustly for both MUAe amplitude and MUAe slope (Fig. 5Q, Fig. S5B,C).

## DISCUSSION

In this study, we investigated travelling waves along the cortical depth using LFP and MUAe recordings from macaque visual cortical areas V1 and V4. We found that LFP TWs are robustly organized across depth and modulated by sensory experience. Although no TWs were observed in MUAe, we detected significant MUAe synchrony during LFP TWs, indicating coherent computations across layers. Importantly, LFP TW speeds predicted the dynamics of population activity: faster waves were associated with a sharper rise and larger peaks of MUAe. Finally, for a significant period of LFP TWs, both spike probability and MUAe signaled lowered excitability compared to periods of no TWs. These observations motivate a mechanistic framework in which LFP TWs reflect the volume-conducted signature of spatiotemporally structured synaptic inputs along the cortical depth, while MUAe reflects the leaky integration of those same inputs within the cortical columns. Predictions derived from this framework were supported by electrophysiological data across sensory conditions and cortical areas. In contrast to propagation-based models, our framework predicts that manipulations of the temporal structure of inputs along the cortical depth, including changing sensory drive, should systematically modulate TW velocity and the associated population response, as observed in our data (Fig. 2D). Together, these results indicate that TWs along the cortical depth reflect structured synaptic input dynamics rather than the transmission of spiking activity.

### Interpretation of framework

The central implication of our framework is that the temporal organization of structured synaptic inputs, rather than propagating spike activity, determines both the dynamics of laminar LFP TWs and cortical excitability. A key feature of this framework is that the same spatiotemporal organization of predominantly excitatory inputs gives rise to LFP TWs while, through leaky integration, determining the resulting population spiking. Faster traveling waves correspond to more temporally aligned inputs, producing larger integrated depolarization and stronger population spiking, whereas slower waves reflect greater temporal dispersion and weaker responses. Within this framework, MUAe provides a particularly informative readout because it reflects the integrated output of the local neuronal population rather than sparse activity from individual neurons. Combined with the broader spatial sampling of LFP through volume conduction, these complementary signals permit direct comparison between the spatiotemporal organization of synaptic inputs and the resulting population output. Because the spatiotemporal organization of synaptic inputs varies across individual trials, averaging would obscure both their transient organization and their relationship to cortical output. Single-trial analyses are therefore essential for revealing the link between LFP TWs and neuronal population responses.

Our observations are most parsimoniously explained by structured, and predominantly excitatory, inputs. Within the laminar cortical network, these structured inputs could arise from distinct patterns of spatiotemporal organization of long-range excitatory pathways conveying information about the sensory environment. This interpretation predicts systematic differences in TW speeds and excitability across sensory conditions, consistent with our observations during dark and visual stimulation (Fig. 1,2). Internal brain states may similarly reshape the spatiotemporal organization of inputs within a sensory condition, providing a potential explanation for the correlated fluctuation of LFT TW speed and MUAe dynamics observed during the dark period (Fig. 4K,L).

Although we illustrated the framework using simplified current ensembles (Fig. 5A), the mechanism is not restricted to these configurations. More complex patterns — including delayed activation of multiple pathways targeting local neural populations or common pathways innervating multiple depth locations^52–54^— can also generate LFP TWs (Fig. S5A) while preserving the relationship between TW speed and MUAe dynamics. Rapid activation of a feedforward chain along the cortical depth^63^ could also produce spatially separated dipoles causing a detectable TW in LFPs but not in MUAe. However, unlike our framework, such mechanisms do not naturally account for the observed relationship between TW speed and MUAe dynamics.

A key implication of our framework is that LFP TW speed provides a proxy for the temporal delays between distributed synaptic inputs. Volume-conducted LFP TW speeds can reflect the interplay of spatial separation between the current sources/sinks in the ensemble, their relative magnitude, temporal dynamics, and temporal offsets^40,50^. Unlike spatial separation, which is constrained by cortical thickness, temporal delays can be dynamically tuned by factors including presynaptic release probability^64^, balance of subthreshold currents^65,66^, and network dynamics^67,68^ over a wide range of values. Consequently, manipulations that selectively alter the temporal organization of synaptic inputs should systematically change traveling-wave speed and the associated population response. These predictions could be tested directly using techniques such as holographic optogenetics to deliver spatiotemporal controlled patterns of input ensembles and measuring the resulting spatiotemporal LFP and MUAe activity^69^.

Importantly, the framework does not imply that synchronized population activity requires TW-generating input patterns. Other mechanisms, such as a strong feedforward drive in a laminar network, can synchronize population activity without producing coherent LFP TWs. This distinction may explain the weaker correlation between LFP TWs and MUAe synchrony during the post-response period in our data, when visually-evoked feedforward activity dominates in the visual cortices (Fig. S4B).

### Relationship to previous works

Computational modeling has shown that LFP TWs can emerge in spiking neural networks (SNNs) with conduction delays^60,61^, in which LFPs are reconstructed using volume conduction models^70^. Our framework is complementary to these approaches, as it addresses what the resulting LFP traveling waves represent physiologically. In principle, structured synaptic current ensembles could also emerge within SNNs, providing a potential link between these frameworks (Fig. 5A and Fig. S5A). More broadly, our results suggest that traveling waves may involve complementary mechanisms across spatial scales. Large-scale anatomical connectivity could shape TW geometry, while the spatiotemporal organization of local synaptic inputs could shape TW speed and neuronal excitability.

Other mechanisms, such as ephaptic coupling^51,71^, have also been proposed for traveling waves. While ephaptic interactions may contribute under some conditions, their reported modulation^72^ appears insufficient for explaining the substantial changes in firing rates as observed here. Our results instead suggest that structured synaptic inputs provide a parsimonious explanation for both the observed LFP traveling waves and their relationship to neuronal output.

Prior studies have linked surface TWs to cortical excitability^14,26,49^. Consistent with these findings, we found that depth TWs were related to both spike probability and MUAe phase, and extend this work by providing a mechanistic framework for this association. Within this framework, trial-to-trial variability in TW organization reflects fluctuations in the temporal structure of synaptic drive, shaping the magnitude of sensory-evoked responses^14^. This interpretation makes a testable behavioral prediction: in near-threshold sensory change detection tasks, where success and failure are equally probable, TW speed should predict subsequent performance because it reflects trial-to-trial, state-dependent fluctuations in the timing of sensory input with respect to cortical excitability.

Studies have also shown that TW direction changes with sensory experiences^15,20^, motor planning^17,23,24^, and stages of a working memory task^13,26^. Consistent with these findings, we observed a reversal in the dominant direction of LFP TWs before and during visual stimulation (Fig. S2A). Our framework suggests that TW direction and TW speed encode complementary aspects of cortical processing. Whereas direction may indicate which pathways are preferentially recruited (e.g., feedforward versus feedback across visual conditions), TW speed reflects the temporal organization of the recruited synaptic inputs and therefore predicts their impact on neuronal activity. Consistent with this interpretation, TWs in either direction were correlated with MUAe synchrony (Fig. S2B), and empirical data from both sensory conditions supported the framework, indicating that the same synaptic integration mechanism applies regardless of the propagation direction.

This distinction between TW direction and speed provides a new perspective on theories that assign computational roles to traveling waves. TWs have been proposed to play a functional role in predictive processing^73^, a theory proposing that the brain examines incoming sensory information against its predictions; feedback pathways carry sensory predictions while feedforward pathways carry prediction error. Human EEG studies have reported stimulus-dependent switch in TW direction, suggesting that opposing TW directions mediate prediction and error signaling^15^. However, subsequent studies^74^ show a mixed support for this interpretation. Instead of assigning a role solely to TW direction, our framework offers a complementary mechanistic perspective: prediction error could modulate the spatiotemporal organization of feedforward and feedback inputs, altering TW speed, and hence cortical excitability. Whether TW speed systematically encodes prediction error is a directly testable hypothesis.

TWs in spiking activity have been reported in several systems^40,75–77^. However, these findings have largely relied on trial averaging, or analytical framework that differs between the field potentials and spiking activity. By applying the same analytical framework for both LFP and MUAe signals, we directly compare the spatiotemporal patterns reflecting synaptic inputs and population outputs, and reveal a dissociation that is obscured by averaging. This is consistent with our proposal that LFPs and MUAe report different stages of the same underlying synaptic process.

Human and rodent studies further suggest that TW patterns reflect the anatomical organization in the cortex^49,78^. Recent cortex-wide calcium imaging demonstrated that rotating TWs on the cortical surface are shaped by axonal geometry^49^. Our findings address a complementary level of explanation by showing how laminar TWs are transformed into neuronal output within local cortical circuits. Together, these studies suggest that anatomy may determine the spatial organization of traveling waves, whereas local synaptic integration could govern their downstream impact on neuronal output.

Using laminar recordings from macaque cortex, we show that LFP TWs are dissociated from population spiking, yet predict the dynamics of neuronal responses. We propose a mechanistic framework in which the LFP TWs reflect spatiotemporally structured synaptic inputs, while population spiking reflects their leaky integration within cortical columns. By providing a physiological interpretation of LFP TWs along the cortical depth, this framework links extracellular field dynamics to neuronal output while generating experimentally testable predictions. More broadly, our findings suggest that TW speed provides a readout of the organization of synaptic inputs, offering a new perspective on how cortical activity is coordinated across space and time.

## METHODS

### Surgical procedures

Surgical procedures have been described in detail previously^56,79^. In brief, low-profile titanium recording chambers were implanted in two male rhesus macaques (Macaca mulatta; M1: age 6 years, M2: age 8 years), providing access to visual areas V1 and V4 (right hemisphere of Monkey M1, bilaterally in Monkey M2). Following implantation, the native dura mater was surgically removed and replaced with a transparent silicone artificial dura (AD), which enabled direct visualization of V1 and V4 cortical sites for precise probe targeting. All procedures were approved by the Yale University Institutional Animal Care and Use Committee and conformed to NIH guidelines.

### Electrophysiology

Electrophysiological data were collected using linear array (‘laminar’) probes (64-channel electrode arrays, NeuroNexus Technologies, Inc.; 2 shanks, 32 channels per shank, 70 μm inter-channel spacing, 200 μm inter-shank spacing) that were inserted perpendicular to the cortical surface. For each monkey, recordings were obtained separately from V1 and V4. In our analysis, each shank’s data was treated as an independent recording session. Further details regarding electrophysiology are provided in Xu, Morton et al., 2024 and Morton, Denagamage et al., 2025^56,79^.

### Extraction of LFP, MUAe, and spiking activities

Electrical signals from the laminar probe were sampled at 30 kHz, digitized via a 64-channel digital headstage, and transmitted to the recording system (RHD Recording System, Intan Technologies). The LFP signal was extracted by bandpass filtering the wideband 30 kHz signal between 4 and 200 Hz and down-sampling to 1 kHz. Spiking activity was extracted offline using Kilosort2 and manually sorted into single- and multi-unit clusters (see Xu et al., 2024 and Morton, Denagamage et al., 2025^56,79^ for more details). The MUAe signal was computed by bandpass filtering the wideband signal between 500 and 9000 Hz, rectifying, low-pass filtering at 200 Hz, and down-sampling to 1 kHz^80^.

### Experimental task

During the dark period, the monkeys sat in a dark room (non-scotopic conditions; luminance in the recording room was 0.054cd/m2 measured using a Sekonic L-478D-U photometer) for 10–40 minutes while making voluntary saccades. During the visual stimulation period, the monkeys maintained fixation at the center of a gray calibrated monitor while stimulus arrays were presented for 100 ms at 200–250 ms intervals. The arrays consisted of Gabor patches (one or two patches) that were presented at or near (within 1.25 degrees of visual angle) the center of the receptive field (RF), which was taken as the center of the average of the RFs across all recording sites and was determined by a separate reverse-correlation-based mapping procedure at the start of each session. The specific arrangement of the stimulus arrays was not relevant to this study, and data were aggregated across all arrangements for all further analyses. Additional details on visual stimulation protocols are available in Xu, Morton et al., 2024 and Morton, Denagamage, et al., 2025^56,79^.

### Channel inclusion criteria

Channels within 500μm of the boundaries of the input layer, or those with valid single-unit or multi-unit activity, were included in the analysis. All channels between the top-most and bottom-most valid channels (as defined above) were also included. The laminar boundaries were identified using standard CSD mapping procedures^56,79,81^, with the input layer defined by the boundaries of the earliest current sink that characterizes feedforward input into layer IV.

### Traveling wave and synchrony detection

We adapted established methods^14^ to identify spatiotemporal patterns along cortical depth (Fig. 1A, Fig. 4A). The first step in both pipelines— detecting traveling waves and identifying synchrony—was to calculate the generalized phase.

A 4th-order 5–40 Hz Butterworth filter was applied to the signal, minimizing distortion and filter artifacts^14^. The Hilbert transform was then used to obtain an analytic signal representation. The analytic signal’s generalized phase ϕ (GP) captures the dominant temporal fluctuation on each channel^82^, providing a phase representation that reduces noise through temporal convolution. Traveling waves and synchrony patterns were detected using distinct spatial and temporal coherence criteria.

For traveling wave (TW) detection, we focused on linear TWs of the form ϕ=kx-ft, where k is the spatial frequency and f is the temporal frequency. We computed the spatial phase gradient $\nabla_{x}\phi$, and averaged across channels to obtain the phase gradient directionality, $\rho =\frac{\bar{\nabla_{x}\phi }}{|\bar{\nabla_{x}\phi |}}$, where the overline denotes channel averaging. |ρ| ranges from 0 to 1 and quantifies spatial coherence^44^; consistent phase gradient signs yield ρ near 1. Significant spatial coherence was defined as |ρ|>0.6, exceeding 99% of ρ values compared to white noise. Significant temporal coherence required the spatial coherence last for at least 30 ms while maintaining the sign of ρ. The 30 ms duration ensured TW probability was not overly reduced by the stricter criterion, while maintaining robustness against noise^83^ (Fig. S1).

For synchrony detection, phase alignment was measured by the resultant vector length $R=|\bar{e^{i\phi }}|$, where the overline indicates channel averaging. R ranges from 0 to 1 and approaches 1 when phases are aligned^59^. Significant spatial coherence was defined as R>0.6, above 99% of R values from white noise. Significant temporal coherence required R>0.6 for at least 30 ms. With R>0.6, the standard deviation of the phase during MUAe synchrony was approximately ±8.7 ms after normalizing by the channel-averaged frequency (Fig. S4C).

### Definition of dark, pre-response, and response periods

For quantitative analysis, three periods corresponding to distinct sensory experiences were defined: dark, pre-response, and response. Dark period ‘trials’ began 100 ms after saccade offset and ended 50 ms before the next saccade onset, excluding trials shorter than 250 ms. We then removed the first and last 30 ms of each dark trial to avoid edge distortion. This criterion minimized potential saccade-related artifacts while maximizing the amount of usable data. The pre-response and response periods were defined as −100 to 0 ms and 50 to 150 ms relative to stimulus onset, respectively. To minimize edge distortion, these specific windows were extracted from a broader analysis epoch spanning −200 to 250 ms relative to stimulus onset.

### Traveling wave speed Estimation

For each time point with a detected TW, TW speed was estimated as the ratio of average temporal and spatial phase gradients: $|v|=\left|\frac{\frac{\bar{\partial \phi }}{\partial t}}{\frac{\partial \phi }{\partial x}}\right|$.

### Temporal evolution of traveling wave probability and speed

To visualize the temporal evolution of LFP TW, we defined traveling wave (TW) probability at a given time point as the percentage of time-locked trials exhibiting a TW. Similarly, we calculated TW speed at each time point as the mean speed across all time-locked trials where a TW was detected. During the dark period, the trials are defined as 430 ms to 770 ms after saccade onset. During the stimulation period, stimuli were presented in blocks of 4 – 6^56,79^. To illustrate the temporal evolution over three flashes, trials were defined as from 170 ms before to 1002 ms after the onset of the first stimulus of the block.

### Existence of traveling waves and synchrony

To quantitatively assess the existence of TW or synchrony patterns for a given sensory experience period, we compared the observed probability to those obtained from spatially and temporally shuffled data. For spatial shuffling, a probability distribution was generated by shuffling channel identities 20 times. For temporal shuffling, the time series of each trial was shuffled 20 times. One-tailed t-tests determined whether the observed probability was significantly higher than the shuffled distributions. P-values $p_{\text{space}}$ and $p_{\text{time}}$ were obtained for spatial and temporal shuffles, respectively. TW was considered significant if both $p_{\text{space}}$ and $p_{\text{time}}$ were less than 0.05. Synchrony was considered significant if $p_{\text{time}}$ was less than 0.05 because spatial shuffling does not affect synchrony patterns.

### Spike probability analysis

To compute average spike probability for a given speed, all LFP TW speeds were divided into ten bins based on the LFP TW speed distribution with spikes. Ten evenly spaced bins were created, covering the range from the mean LFP TW speed during spikes minus twice the standard deviation to the mean plus twice the standard deviation. This included about 95% of the data points. Sessions with fewer than 100 spikes during the periods of interest were excluded to ensure sufficient data. Speed bins with fewer than 10 spikes were excluded from average spike probability calculations. Spike probability was calculated as the total number of spikes across all channels in each speed bin, normalized by the total number of single units detected and the total duration of LFP TWs in that bin. Baseline spike probability was defined as the spike probability during periods without TWs. The percentage of time points below or above baseline was calculated by identifying speed bins with spike probability below or above that of the no LFP TW baseline. The percentage was computed as the timepoints captured by those bins divided by the total timepoints captured by all the bins.

For LFP TW periods with or without MUAe synchrony, spike probability was calculated as the total number of spikes across all channels for the entire period, normalized by the total number of single units detected and the total duration of that period.

In all spike probability analyses, bursts of spikes within 1 ms on a single channel (a rare event) were counted only once.

### Average MUAe and average MUAe phase

During the periods of synchrony, the average MUAe is calculated as the average of raw MUAe across the channels. The average MUAe phase is calculated as the absolute value of the circular mean of the MUAe phase across the channels. We took the absolute value after taking the average for the ease of analysis because the generalized phase framework suggests that the same absolute phases represent the same MUAe magnitudes. This does not affect our conclusions. Note that the average MUAe phase is only well defined for periods with MUAe synchrony.

### Pointwise mutual information

Pointwise mutual information^84^ was used to quantify the correlation between LFP TWs and MUAe synchrony. It is defined as the probability that LFP TWs and MUAe synchrony co-occurred, divided by the probability that they would co-occur if they were independent events: $PMI=log\frac{prob(LFP\;TW\;\&MUAe\;\text{Sync})}{prob(LFP\;TW)\;\text{prob}(MUAe\;\text{Sync})}$. PMI approaches zero if the two events are independent.

### Coefficient of variance

The coefficient of variance (CV) is calculated as the ratio of the standard deviation to the mean. The ΔCV is the difference between the average CV during periods of LFP TW and the average CV when there is no LFP TW.

### Seasonal Kendall test

To control for MUAe phase when calculating the correlation between LFP TW speed and MUAe, we binned the MUAe phases into ten equal intervals from 0 to 2π, ensuring a minimum of 10 data points per bin. We then applied a seasonal Kendall test^85^, treating each phase bin as a distinct “season,” to assess the significance of the correlation between TW speed and MUAe.

### CSD Models

To demonstrating core concepts of the proposed TW mechanism, we modeled unipolar current sink ensembles for simplicity (for dipole configurations such as those shown in Fig. S5A, see below.). To represent the spatiotemporally diffuse current sinks corresponding to columnar inputs at depth x, we modeled the current source density (CSD) of a unipolar current (Fig. 5H) as follows^40,50^:

$$CSD\left(x,t\right)=-\left[t\cdot \exp\left(-\frac{t^{2}}{2\tau_{s}^{2}}\right)\cdot u\left(t\right)\cdot \exp\left(-\frac{x^{2}}{2\sigma_{x}^{2}}\right)\right]\;*\;\left[A\cdot \delta \left(x-X_{i},t-T_{i}\right)\right]$$

Here, A represents the strength of the excitatory inputs targeting the cortical column (Fig 5A), which is assumed to be equal for simplicity. $\tau_{s}$ represents the timescale of currents, $\sigma_{x}$ represents the spatial extent of currents, $X_{i}$ represents the depth at which input i arrives, and $T_{i}$ represents the time at which input i reaches the cortical column. The Heaviside function u ensures causality and the relatively brief activation of inputs is captured by the δ function.

The time delay between a unipolar current sink/source pair is defined as the difference between their onset times:

$$\tau =T_{2}-T_{1}.$$

Input triggered dipoles (Fig. 5D, S5A) were similarly modeled:

$$CSD(x,t)=-\left[t\cdot exp\left(-\frac{t^{2}}{2\tau_{s}^{2}}\right)\cdot u(t)\cdot exp\left(-\frac{x^{2}}{2\sigma_{x}^{2}}\right)\right]*\;\;\left[A\cdot \delta \left(x-X_{i+},t-T_{i}\right)-A\cdot \delta \left(x-X_{i-},t-T_{i}\right)\right]$$

where $X_{i+/-}$ represents the depth at which the centers of positive and negative moments of the dipole are located.

### Volume Conduction Model

The LFP was computed from the CSD using a volume conduction model^50,86^, which assumes that field potentials represent the spatiotemporal sum of both nearby and distant synaptic currents:

$$LFP(x,t)=R_{\text{tissue}}\sum_{k=1}^{N}\frac{{CSD}_{k}(x,\;t)}{\sqrt{h^{2}\;+\;{\left|x\;-\;x_{k}\right|}^{2}}}.$$

Here, $R_{\text{tissue}}$ represents tissue resistivity, h is the horizontal distance between the recording channel and the CSD source which we chose to be a non-zero value, and $\left|x-x_{k}\right|$ denotes the vertical distance between the channel and the CSD source at position $x_{k}$. The denominator represents the total physical distance from the CSD source or sink to the channel.

We applied the same analysis pipeline used for the experimental data to detect LFP TWs and calculate their propagation speeds (Fig. 1A). For each time delay τ, we estimated the LFP TW speed by averaging across all detected LFP TWs within a time window from the onset of the first input to 50 ms after the onset of the second.

### MUAe Model

We modeled the multi-unit activity envelope (MUAe) as reflecting a combination of excitatory inputs targeting the entire cortical column (${\mathrm{MUAe}}_{\mathrm{global}}$, Fig 5A) and independent local spiking activity (${\mathrm{MUAe}}_{\mathrm{local}}$):

$${MUAe}_{\mathrm{channel}}(x,t)={MUAe}_{\mathrm{global}}(x,t)+{MUAe}_{\mathrm{local}}.$$

${\mathrm{MUAe}}_{\mathrm{global}}$ was modeled as a leaky integrator, while ${\mathrm{MUAe}}_{\mathrm{local}}$ was modeled as Gaussian noise. For ${\mathrm{MUAe}}_{\mathrm{global}}$, we considered the simplest scenario where a pair of globally shared inputs were combined through leaky integration followed by threshold nonlinearity:

$$\;{MUAe}_{\mathrm{global}}(x,t)={\left[u_{\mathrm{global}}(t)-\theta +\varepsilon_{\theta }(x)\right]}_{+}\;u_{\mathrm{global}}(t)=\left[t\cdot exp\left(-\frac{t}{\tau_{\mathrm{int}}}\right)\cdot u(t)\right]\;*\;\left[A\cdot \delta \left(t-T_{1}\right)+A\cdot \delta \left(t-T_{2}\right)\right]$$

Here, A and $T_{i}$ represents the strength and onset times of the columnar inputs as in the CSD model. $\tau_{\mathrm{int}}$ represents the timescale of columnar leaky integration resulting from a combination of membrane properties and recurrent network connectivity in the column. The Heaviside function u ensures causality and relatively brief activation of inputs is captured by the δ function. θ is a threshold that captures a spiking nonlinearity. $\varepsilon_{\theta }$ represents channel-specific Gaussian noise that effectively modifies the threshold θ and is fixed in time. Note that the spatial variation of the ${MUAe}_{\mathrm{channel}}(x,t)$ comes from ${MUAe}_{\mathrm{local}}$ and $\varepsilon_{\theta }$.

For the simple case of equal input strengths and $t>T_{2}$,

$$u_{\mathrm{channel}}(t)=A\;exp\left(-\frac{t-T_{1}}{\tau_{\mathrm{int}}}\right)[\left(t-T_{1}\right)\cdot \left(1+exp\left(\frac{\tau }{\tau_{\mathrm{int}}}\right)\right)-\tau \cdot exp\left(\frac{\tau }{\tau_{\mathrm{int}}}\right)].$$

Thus, both the peak time and peak amplitude of MUAe in our model depend on the time delay between the two inputs ($\tau =T_{2}-T_{1}$), as illustrated in Fig. 5I and Fig. 5N. To simulate weak input (Fig. 5O), we reduced the input magnitude A to half of the standard value.

Our model simulation results were robust across different parameter choices, alternative nonlinear functions (e.g., sigmoid-type exponential nonlinearity), and varying noise levels.

### Model parameters

**Table:** 

Symbol	Value

A	100
$\sigma_{x}$	140
$\tau_{s}$	30
$R_{\text{tissue }}$	0.16
h	158
$\tau_{\text{int }}$	40
θ	1500
${\mathrm{MUAe}}_{\mathrm{local}}$	0.2×𝒩(0,1)
$\varepsilon_{\theta }$	500×𝒩(0,1)
τ	20–40

## Supplementary Material

Supplement 1

## Figures and Tables

**Table 1.** Session and channels counts for the figures.

Animal region	M1 V1			M2 V1			M1 V4			M2 V4		
Sensory experience periods	dark	pre-resp	resp	dark	pre-resp	resp	dark	pre-resp	resp	dark	pre-resp	resp
Fig. 1E	18	16	16	6	24	24	24	22	22	14	6	6
Fig. 3D	16	16	16	6	24	24	24	21	21	8	3	3
Fig. 3E	16	16	14	6	17	23	22	20	18	7	2	3
Fig. 3F	16	16	16	6	24	24	24	21	21	8	3	3
Fig. 4C	18	16	16	6	24	24	24	22	22	14	6	6
Fig. 4D	18	16	16	6	24	24	24	22	22	14	6	6
Fig. 4E, S4B	14	10	16	6	17	24	24	22	22	12	4	4
Fig. 4G	13	10	16	6	17	24	24	22	22	11	3	3
Fig. 4H	13	9	16	6	16	24	24	22	22	10	3	2
Fig. 4I	13	10	16	6	17	24	24	22	22	11	3	3
Fig. 4L	13	12	18	6	17	24	24	22	22	10	3	3
Fig. 5H	18	16	16	6	24	24	24	22	22	13	5	6
Fig. 5L	14	12	18	6	17	24	24	22	22	12	4	4
Fig. 5M	13	11	17	6	14	24	24	22	22	11	3	3
Fig. 5P	12	12	18	6	17	24	24	21	21	12	3	3
Fig. 5Q, S5B,S5C	395	330	498	192	480	664	742	668	668	358	112	112
Fig. S2B	12	8	14	6	17	24	24	22	22	12	4	4
Fig. S3A Broad Spiking	16	16	16	6	22	22	22	21	21	6	3	3
Fig. S3A Narrow Spiking	16	16	16	5	23	23	21	17	18	4	1	1
Fig. S3B Superficial Layer	11	12	12	6	17	19	13	14	14	6	3	3
Fig. S3B Input Layer	15	15	15	6	22	22	20	21	21	7	2	2
Fig. S3B Deep Layer	14	15	16	6	22	22	22	20	20	2	1	1
