# Supplementary material for: Traveling waves along the cortical depth reflect structured synaptic inputs
Source: Res Sq. 2026 Aug 26:rs.3.rs-10385703. Preprint. [Version 1] doi: 10.21203/rs.3.rs-10385703/v1 (PMC13532707; doi:10.21203/rs.3.rs-10385703/v1)
Supplement: Supplementary file 1 [file NIHPPRS10385703v1-supplement-1.pdf]

Supplementary Figures

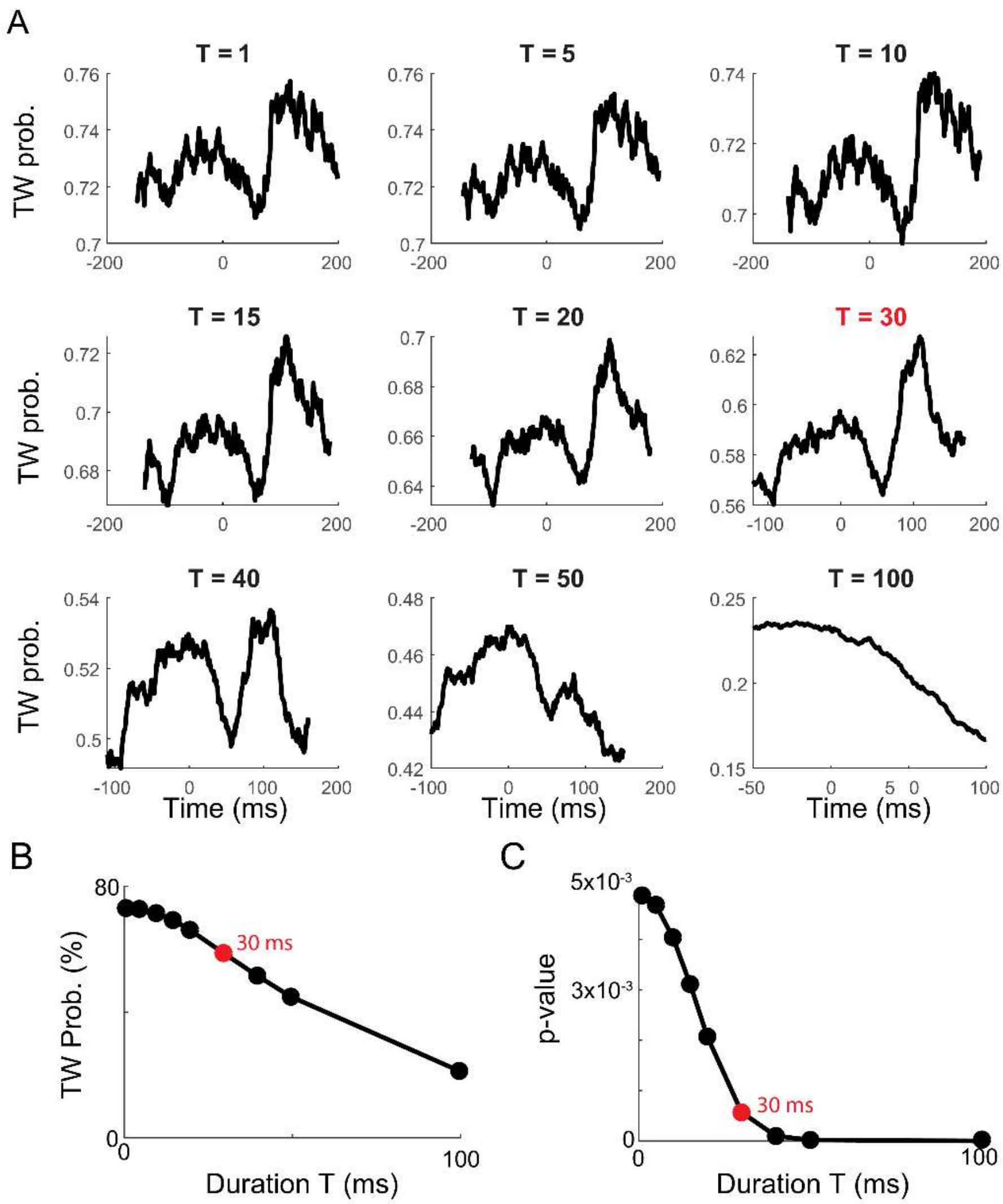

Figure S1. Temporal duration criterion affects the LFP traveling wave probability and analysis robustness based on a single session. (A) Temporal evolution of traveling wave probability with different duration criteria (T). Stimulus onset is at 0 ms. The 30 ms duration used in the analysis pipeline is marked in red. (B) Average TW probability for different T. (C) The p-value when using different duration criteria (T). The alternative hypothesis is that the TW probability in the session is higher than in

temporally shuffled controls (one-tailed t-test, the LFP signal is shuffled 5 times).

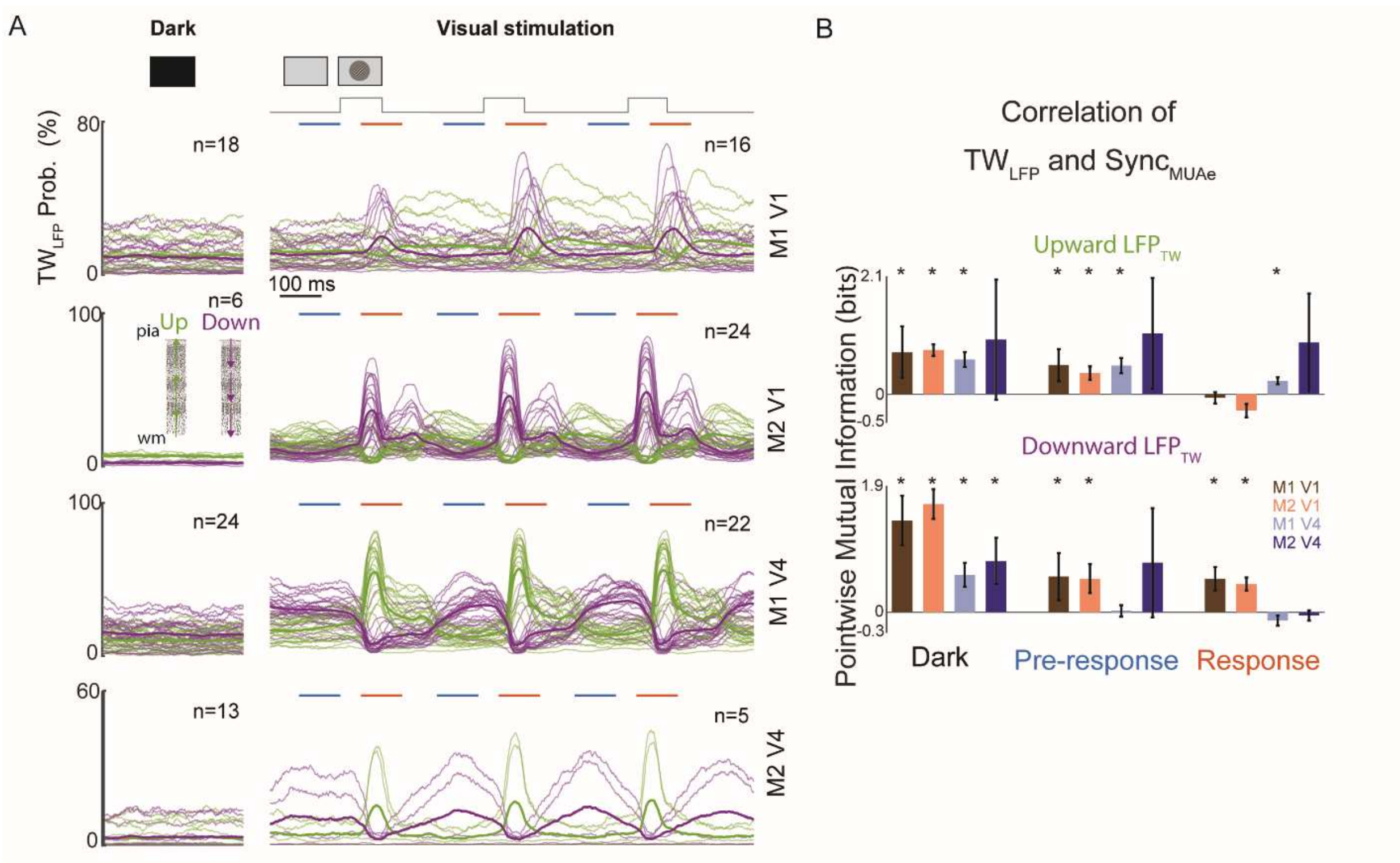

Figure S2. Modulation of upward and downward LFP traveling wave probability during Gabor stimulation and LFP TW correlation with MUAe synchrony. (A) Temporal evolution of LFP TW probability for different directions. Thick line represents session-averaged probability and thin lines represent single sessions. Blue and red bars mark the pre-response and response periods, respectively. *n* indicates the number of sessions. (B) Correlation between MUAe synchrony and LFP traveling wave when

separated by directions. $p < 0.05$, t-test. Error bars indicate 95% confidence intervals.

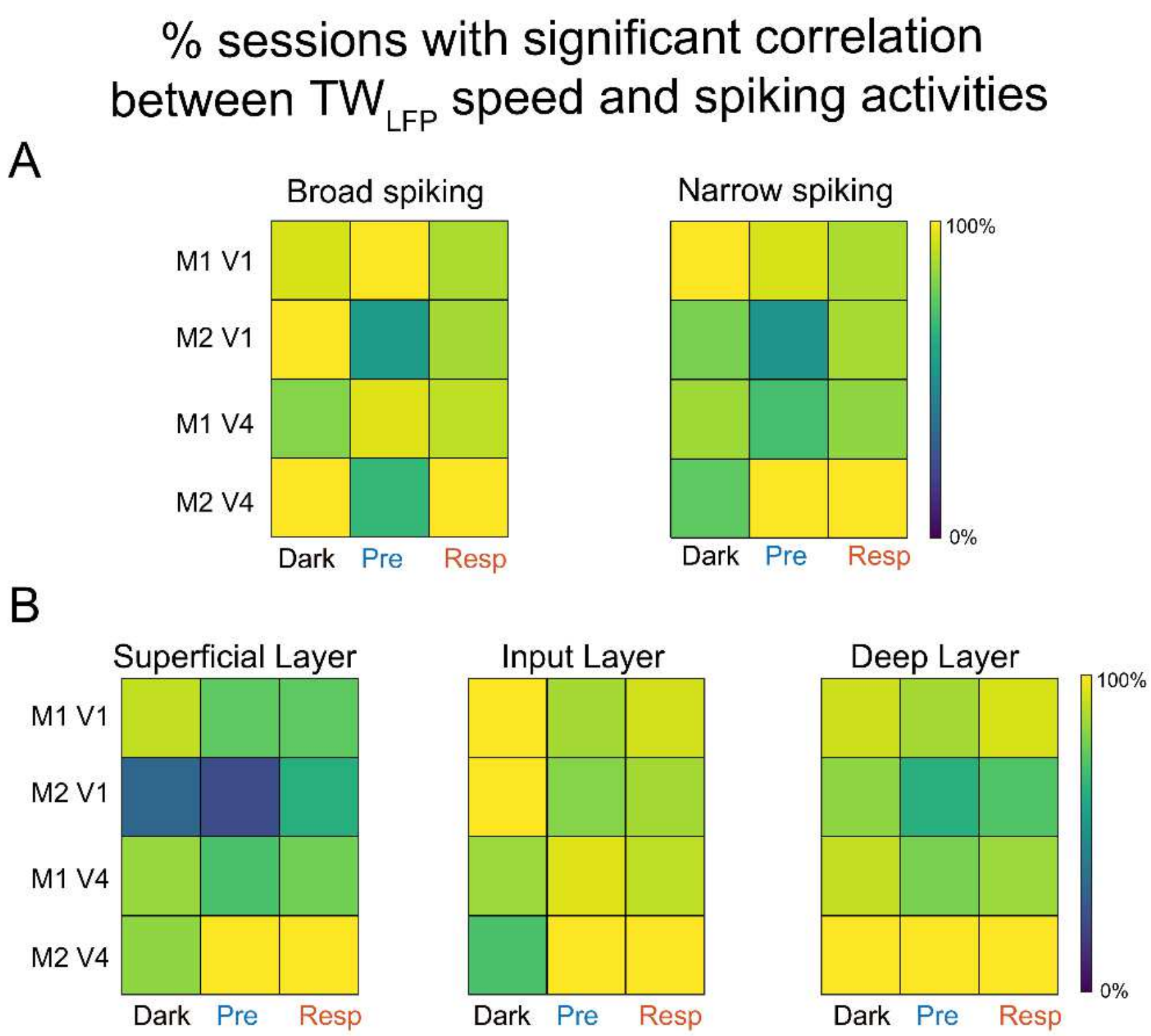

Figure S3. Percentage of sessions with significant correlation between LFP traveling wave speed and spiking activities when separated into different cell types and layers. (A) Separated by waveform profiles. Neurons with waveform durations less than 350μs were classified as narrow-spiking, while those with waveform durations longer than 350μs were classified as broad-spiking. (B) Separated by cortical layers. Current source density (CSD) analysis[1] was used to estimate the boundaries between the superficial (I--III), input (IV), and deep (V--VI) cortical layers, and to assign individual neurons their layer identity[2]. See Morton 2025[3] for further methodological details regarding cell classification and layer assignment.

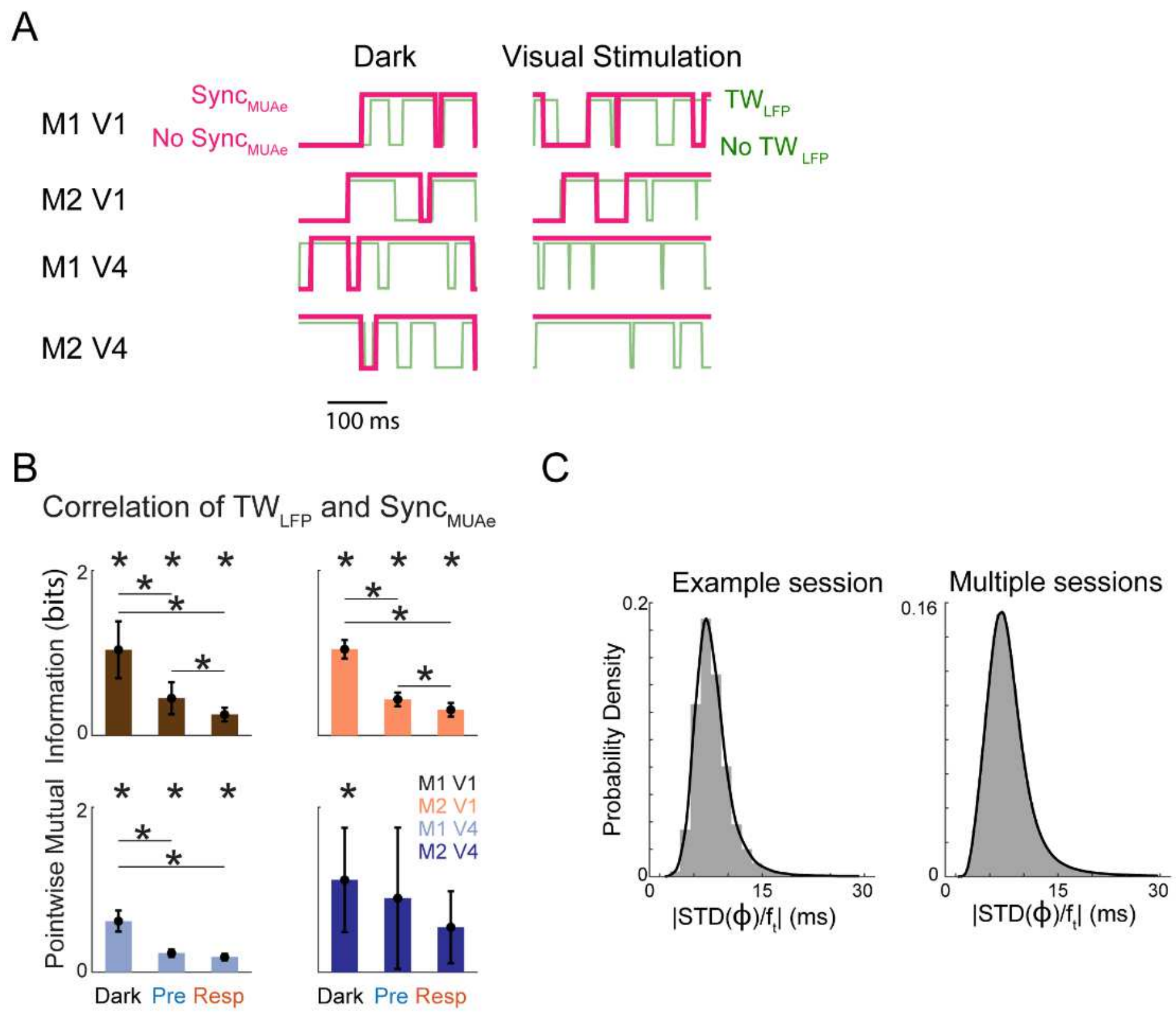

Figure S4. Correlation of LFP traveling wave and MUAe synchrony and MUAe temporal variation during synchrony. (A) Example trials demonstrating the co-occurrence of LFP TWs and MUAe synchrony. (B) Correlation between MUAe synchrony and LFP TW. The same plot as Fig. 4E but grouped by the brain areas. Error bars are 95% confidence intervals; $p < 0.05$, t-test. (C) Distribution of the estimated variation of MUAe phase during periods of synchrony. The variation is estimated by calculating the standard deviation of the phases across the channels during synchrony and converted to milliseconds by dividing out average temporal frequency f_t across channels. The MUAe phase variation during synchrony could obscure spatial temporal

dynamics on small timescales.

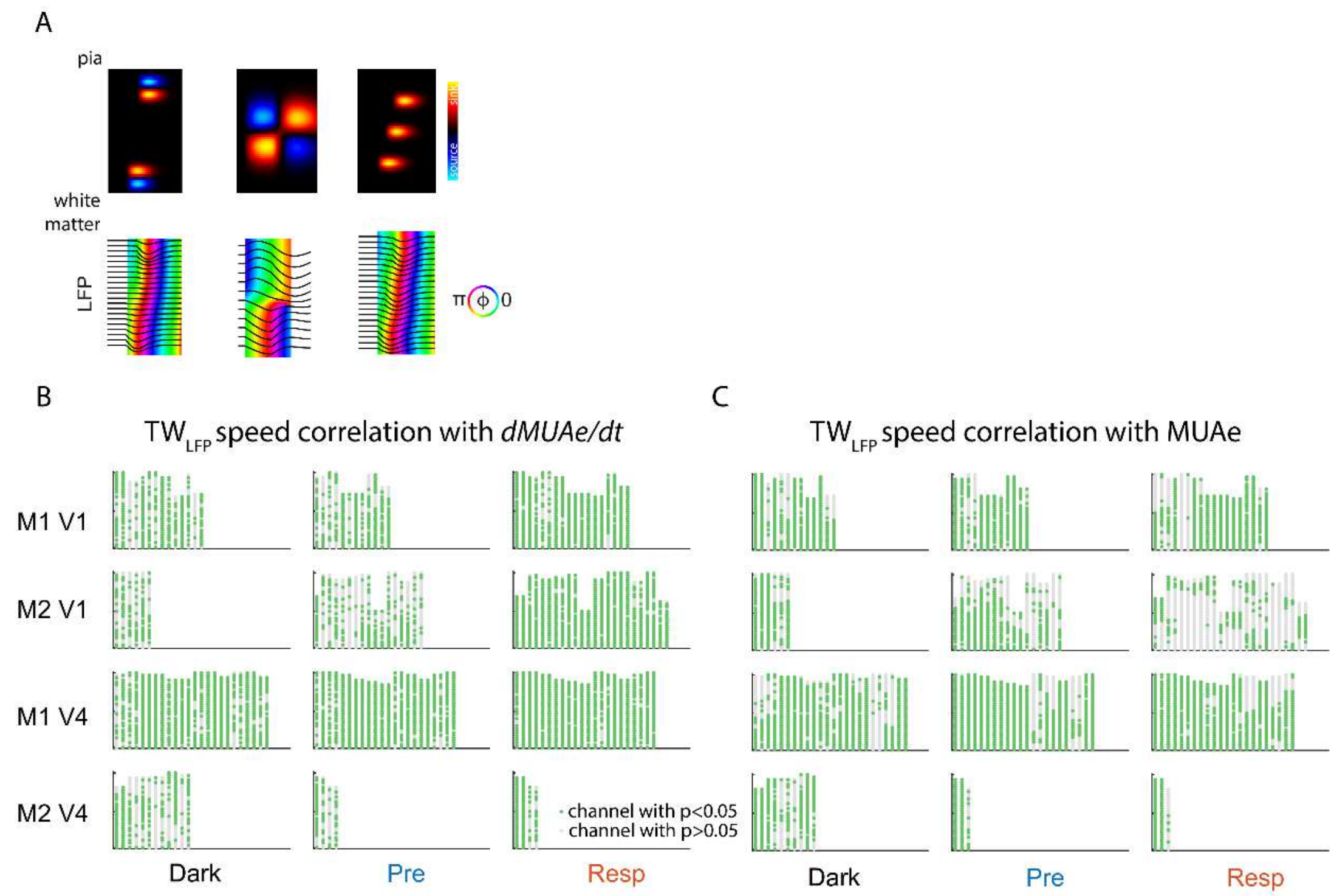

Figure S5. LFP traveling wave could arise from various current source/sink ensemble configurations and are correlated to MUAe even in the absence of synchrony. (A) Complex current sinks/sources could be detected as LFP TWs[1,4]. (B) Correlation of LFP TW speed and *dMUAe/dt* (Pearson correlation). (C) Correlation of LFP TW speed and MUAe magnitude (one-tailed Seasonal Kendall test). For (B) and (C), analyses are at the single-channel level ($p < 0.05$), where each dot represents a channel and each column represents a session. The percentage of channels with significant correlation are summarized in Fig. 5Q.

Table 1. Session and channels counts for the figures.

**Animal region**	M1 V1			M2 V1			M1 V4			M2 V4		
**Sensory experience periods**	dark	pre-resp	resp	dark	pre-resp	resp	dark	pre-resp	resp	dark	pre-resp	resp
Fig. 1E	18	16	16	6	24	24	24	22	22	14	6	6
Fig. 3D	16	16	16	6	24	24	24	21	21	8	3	3
Fig. 3E	16	16	14	6	17	23	22	20	18	7	2	3
Fig. 3F	16	16	16	6	24	24	24	21	21	8	3	3
Fig. 4C	18	16	16	6	24	24	24	22	22	14	6	6
Fig. 4D	18	16	16	6	24	24	24	22	22	14	6	6
Fig. 4E, S4B	14	10	16	6	17	24	24	22	22	12	4	4
Fig. 4G	13	10	16	6	17	24	24	22	22	11	3	3
Fig. 4H	13	9	16	6	16	24	24	22	22	10	3	2
Fig. 4I	13	10	16	6	17	24	24	22	22	11	3	3
Fig. 4L	13	12	18	6	17	24	24	22	22	10	3	3
Fig. 5H	18	16	16	6	24	24	24	22	22	13	5	6
Fig. 5L	14	12	18	6	17	24	24	22	22	12	4	4
Fig. 5M	13	11	17	6	14	24	24	22	22	11	3	3
Fig. 5P	12	12	18	6	17	24	24	21	21	12	3	3
Fig. 5Q, S5B,S5C	395	330	498	192	480	664	742	668	668	358	112	112
Fig. S2B	12	8	14	6	17	24	24	22	22	12	4	4
Fig. S3A Broad Spiking	16	16	16	6	22	22	22	21	21	6	3	3
Fig. S3A Narrow Spiking	16	16	16	5	23	23	21	17	18	4	1	1
Fig. S3B Superficial	11	12	12	6	17	19	13	14	14	6	3	3

Layer												
Fig. S3B Input Layer	15	15	15	6	22	22	20	21	21	7	2	2
Fig. S3B Deep Layer	14	15	16	6	22	22	22	20	20	2	1	1

1. Mitzdorf, U. Current source-density method and application in cat cerebral cortex: investigation of evoked potentials and EEG phenomena. *Physiol. Rev.* **65**, 37–100 (1985).
2. Nandy, A. S., Nassi, J. J. & Reynolds, J. H. Laminar Organization of Attentional Modulation in Macaque Visual Area V4. *Neuron* **93**, 235–246 (2017).
3. Morton, M. P., Denagamage, S., Hudson, N. V. & Nandy, A. S. Non-uniform contextual interactions in the visual cortex place fundamental limits on spatial vision. *Cell Rep.* **44**, 116571 (2025).
4. Mitzdorf, U. & Singer, W. Excitatory synaptic ensemble properties in the visual cortex of the macaque monkey: A current source density analysis of electrically evoked potentials. *J. Comp. Neurol.* **187**, 71–83 (1979).
